# Fertilization Is Not a New Beginning: The Relationship between Sperm Longevity and Offspring Performance

**DOI:** 10.1371/journal.pone.0049167

**Published:** 2012-11-14

**Authors:** Angela J. Crean, John M. Dwyer, Dustin J. Marshall

**Affiliations:** 1 School of Biological Sciences, University of Queensland, Brisbane, Queensland, Australia; 2 Evolution & Ecology Research Centre and School of Biological, Earth and Environmental Sciences, University of New South Wales, Sydney, New South Wales, Australia; 3 School of Plant Biology, University of Western Australia, Crawley, Western Australia, Australia; 4 School of Biological Sciences, Monash University, Melbourne, Victoria, Australia; University of Milan, Italy

## Abstract

Sperm are the most diverse cell type known: varying not only among- and within- species, but also among- and within-ejaculates of a single male. Recently, the causes and consequences of variability in sperm phenotypes have received much attention, but the importance of within-ejaculate variability remains largely unknown. Correlative evidence suggests that reduced within-ejaculate variation in sperm phenotype increases a male’s fertilization success in competitive conditions; but the transgenerational consequences of within-ejaculate variation in sperm phenotype remain relatively unexplored. Here we examine the relationship between sperm longevity and offspring performance in a marine invertebrate with external fertilization, *Styela plicata*. Offspring sired by longer-lived sperm had higher performance compared to offspring sired by freshly-extracted sperm of the same ejaculate, both in the laboratory and the field. This indicates that within-ejaculate differences in sperm longevity can influence offspring fitness – a source of variability in offspring phenotypes that has not previously been considered. Links between sperm phenotype and offspring performance may constrain responses to selection on either sperm or offspring traits, with broad ecological and evolutionary implications.

## Introduction

Given the fundamental role of sperm in reproduction, it is surprising that we lack a comprehensive understanding of the causes and consequences of variability in sperm phenotype [1]. While classic sperm competition theory has focussed on the quantity of sperm that males produce [2], recent attention has turned to the role of sperm quality (sperm phenotype) in postcopulatory sexual selection [3], [4]. Sperm competition and the fertilization environment can exert significant selection pressure on sperm phenotype (such as sperm velocity, sperm viability, and sperm size), and variation among males in sperm phenotype can influence competitive fertilization success [4]. Furthermore, there is growing experimental evidence that males can adjust their sperm phenotype in response to their social environment and perceived risk of sperm competition (e.g. [5], [6], [7]). However, the role of within-ejaculate variability in sperm is less well understood.

Within-ejaculate variation in sperm phenotype is always present, and often substantial [8]; but the adaptive value of this variation remains unclear [9]. All else being equal, postcopulatory sexual selection is expected to minimise variance in sperm phenotype by favouring sperm traits that increase a male’s reproductive success, thus selecting for an optimal sperm phenotype [10], [11]. This expectation is supported by recent studies showing a negative relationship between the risk of sperm competition and within-ejaculate variability in sperm phenotype, suggesting that reduced intra-male variation in sperm phenotype increases fertilization success under competition [8], [12]. However, it is unknown whether these changes in sperm phenotype have any transgenerational effects on offspring fitness. If variability in the phenotype of sperm within males affects offspring fitness, then this would represent an additional selection pressure acting on within-ejaculate variation in sperm phenotype, analogous to maternally-driven offspring size effects on offspring performance. Theory and data show that, because within-brood variation in offspring size can affect maternal geometric fitness, this variation is under strong selection with more variation being favoured when environments are unpredictable [13], [14], [15]. Whether within-male variation in sperm phenotype is under similar selection remains unknown, because the post-fertilization consequences of this variation have not been explored.

External fertilizers provide an excellent system in which to test whether within-ejaculate variability in sperm phenotype has consequences for offspring. Testing the post-fertilization consequences of within-ejaculate variation in sperm phenotype is prohibitively complex in internal fertilizers due to difficulties in manipulating ejaculates without introducing potentially confounding or unrealistic factors. In contrast, external fertilizers are very amenable to *in vitro* fertilization techniques, allowing the easy manipulation of sperm phenotypes in a relatively natural way. Because fertilization takes place externally, rather than in a female reproductive tract, focusing on external fertilizers results in fewer opportunities for females to manipulate which sperm will fertilize their eggs, thus eliminating the confounding influence of cryptic female choice and maternal effects that are likely in internal fertilizers [16], [17], [18]. In this study, we use an externally-fertilizing marine invertebrate, *Styela plicata*, to test whether within-ejaculate differences in sperm phenotype can influence offspring performance. Specifically, we determined whether offspring sired by the longer-lived sperm within an ejaculate differ from offspring sired by freshly extracted sperm within the same ejaculate.

## Materials and Methods

### Study Species and Location

*Styela plicata* is a broadcast spawning (both gametes are shed into the water column) solitary ascidian. It is commonly found growing in a range of densities on man-made structures such as piers, and is considered to be introduced to eastern Australia [19]. Gametes are released in the late afternoon, and larvae hatch the following morning and settle throughout the day. They are protandric hermaphrodites, have a lifespan of <1 year, and are reproductive throughout most of the year (except winter). All animals were collected from, and field work completed at, the East Coast Marina (Manly, Brisbane, Australia; 27.467E 153.183S) - a private access marina which is protected from wave action by a large breakwater. Permission was granted from management of the East Coast Marina to collect animals and complete field work at this site.

### Manipulation of Sperm Phenotypes

We harvested eggs and sperm from separate individuals (which will be referred to as ‘females’ and ‘males’ respectively from herein), using standard strip-spawning techniques [7]. Gonads were dissected from the visceral mass into a Petri dish with a few drops of filtered seawater, and the gonad extract was diced to release gametes. This extract was washed through a 500 µm and then 100 µm filter into a beaker; so excess material was retained in the 500 µm filter, eggs were retained in the 100 µm filter, and sperm was passed through to the beaker. Eggs from three females were pooled together for each fertilization assay, to eliminate systematic differences among the treatments and trials caused by maternal effects and male-by-female interactions. To keep the egg concentration approximately equal among replicates, eggs were mixed and allowed to settle in a beaker, and then 3 mLs of eggs were collected from the base of the beaker for each sample.

To create treatments with different sperm phenotypes, we utilized a split-ejaculate design with time as a selective agent, so that only the average longevity of sperm should differ between treatments. In each trial, 6 ml of sperm extract was collected from a single male; half was used immediately to fertilize eggs (fresh sperm treatment), and the other half was stored in the syringe in a constant temperature cabinet (at 22°C ) for one hour before fertilization (longer-lived sperm treatment). Sperm were stored for one hour as this represents the approximate half-life of sperm in this species [7]. During this storage period sperm were actively swimming, as they are activated while being extracted using the strip-spawning technique. A fresh pool of eggs was used for longer-lived sperm treatment fertilizations, to prevent confounding of egg age with sperm age. Although this method introduced variability into the assays (as offspring within each trial are only paternal half-sibs), there should not be any bias towards either treatment as the pool of eggs was a random sample each time. For both treatments, eggs were rinsed of sperm after 15 mins, and left to develop in 10 ml of filtered seawater in a covered Petri dish.

For each egg sample we estimated fertilization success by scoring all eggs in the field of view (mean = 20) as cleaved or uncleaved when viewed at 30× magnification (5 replicate counts per sample). Fertilization success was calculated 1 h after the initiation of fertilization because more than 50% of cleaved eggs had progressed beyond the 2-cell stage at this time. Sperm concentration was estimated using a Neubauer improved hemocytometer under 400× magnification (3 replicate counts per sample). Although there was a weak positive relationship between sperm concentration and fertilization success (R^2^ = 0.120; F_1,36_ = 4.337; p = 0.044), this relationship was consistent across treatments (treatment×sperm concentration interaction: F_1,36_ = 0.014; p = 0.907), and therefore does not influence the results.

### Effect of Sperm Manipulation on Offspring Size

**Figure 1:**
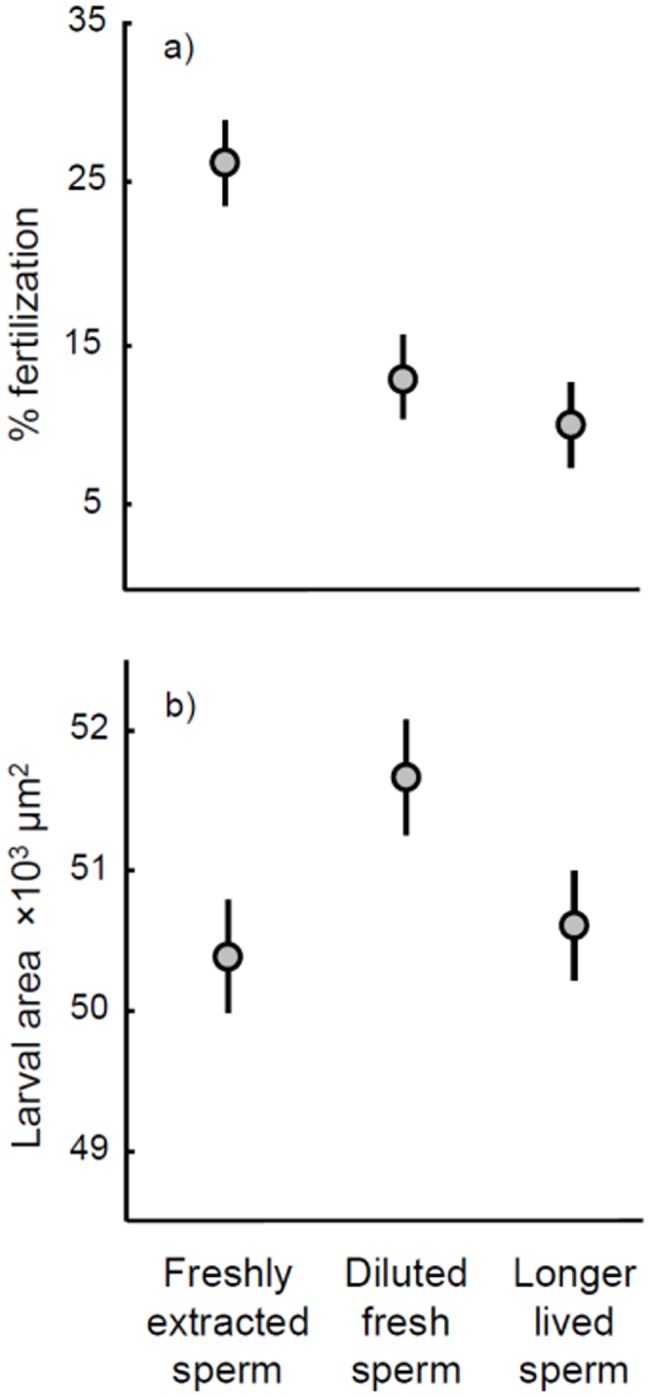
Effect of sperm treatment (fresh sperm, fresh sperm diluted to 1% of the original concentration, and sperm stored for one hour before fertilization) on resulting fertilization success (a); and larval size (measured as area) (b). Points represent least squares mean (± SE).

In broadcast spawners, larger eggs are preferentially fertilized in low sperm concentrations because larger eggs are larger ‘targets’ for sperm and are therefore more likely to come into contact with sperm in sperm limiting conditions [20], [21], [22]. Fertilization assays were deliberately performed in sperm limiting concentrations, so that results were not influenced by polyspermy and all sperm that were capable of fertilization had the opportunity to do so. However, as the effective sperm concentration in the longer-lived sperm treatment is expected to be lower than the fresh sperm treatment (indicated by a drop in fertilization success); we were concerned that the sperm treatment may indirectly influence offspring size. To test for this, we ran a pilot study which included a second fresh sperm treatment which was diluted to 1% of the original sperm concentration. This dilution factor resulted in fertilization success rates similar to those in the longer-lived sperm treatment (Figure 1), and therefore if differences in fertilization success rates are driving differences in offspring traits, we expect to see similar offspring sizes produced from the diluted and longer-lived sperm treatments.

Individual fertilized eggs were collected from each sperm treatment with a micro-pipette and transferred to individual 10 mm diameter wells in a 24-well plate. These plates were stored in a 22°C CT cabinet overnight to develop, and checked every 15 mins the next morning to record time to hatching. As each larvae hatched, it was photographed under 45× magnification using PixeLINK Capture SE software. Larval area was calculated from the digital images by tracing around the perimeter of each larvae using Image-Pro Express (Media Cybernetics). Five replicate trials measuring larval size were completed in Jan to Feb 2009.

### Hatching Success

To estimate hatching success, we collected 20 cleaving eggs (subsamples) from each treatment and left them to develop in 100 ml of filtered seawater at 22°C. Thirteen hours after fertilization, we preserved all larvae and unhatched eggs by adding formalin to each sample jar. Embryos within each subsample were later viewed under 30× magnification and scored as successful (hatched with normal morphology), or unsuccessful (unhatched or hatched with visible deformities). Hatching success was thus calculated as the proportion of fertilized eggs within each treatment that successfully hatched into healthy larvae. Twenty replicate trials measuring hatching success were completed between January to March 2009.

### Post-metamorphic Survival

To estimate post-metamorphic offspring performance, we settled larvae from each treatment into Petri dishes, and transplanted the settlers into the field to measure survival. Eggs were fertilized with fresh or longer-lived sperm using the procedures outlined above, and left overnight to develop in a 250 mL covered beaker in a CT cabinet at 22°C. Successfully hatched larvae were collected with a pipette approximately 12 hours after fertilization, and transferred to a Petri dish for settlement. Larvae were settled into low density (solitary individual) and high density (15 to 30 individuals) treatments, as studies of maternal effects show that the density of conspecifics can strongly affect the relationship between offspring phenotype and offspring performance [23], [24]. In each trial, five low density and three high density offspring treatment replicates were created for each sperm treatment. Larvae were checked to make sure they were free swimming and not stuck in the surface layer, and then the Petri dishes were covered and left undisturbed for 24 hours to allow larvae to settle. Larvae that had not settled within this time (<1% in all replicates) were excluded from our analyses, as we could not distinguish between larvae that could not find a suitable settlement substrate and larvae that were incapable of settlement. Individual settlers were marked and recorded, and Petri dishes transferred to an insulated container filled with filtered seawater ready for transport to the field that afternoon. Petri dishes were suspended vertically within plastic mesh cages (dimensions: 44×28×18 cm length×width×height; mesh size 1 cm^2^) to exclude large predators that were attracted to field equipment. These cages were hung from the pontoons in a relatively high flow area within the marina, approximately 2 m below the water surface. After two weeks, all settlers were transported back to the lab and examined under 30× magnification to measure survival. Nine separate trials measuring post-metamorphic survival were completed in April to May 2009.

### Data Analysis

Fertilization and hatching success were analysed using ANOVA with an unreplicated block design to look at the relative effect of sperm treatment within males. The effect of sperm treatment on larval size was analysed using a 2-factor ANOVA with treatment (fresh sperm, diluted fresh sperm, and longer-lived sperm) as a fixed factor and male as a random factor. The interaction term was not significant (F_8,177_ = 0.741, p = 0.655) and therefore removed from the model. As the effect of treatment was only marginally non-significant, we performed post-hoc tests to look at differences between fresh and longer-lived sperm, and fresh and diluted sperm independently. To analyse the effect of sperm treatment and offspring density on offspring survival, we used mixed-effects logistic regression (Laplace estimation method) with male and dish included as random effects. For the random male effect we allowed the treatment effect of sperm phenotype to vary for each male. Likelihood ratio tests (LRT) were used to assess the significance of treatment effects. We used the LME4 package [25] within the R statistical program [26] to fit all mixed effects logistic models.

## Results

Fertilization success in longer-lived sperm treatments was on average 38% lower than in assays using fresh sperm from the same ejaculate (mean fertilization success ± s.e.: fresh sperm = 37.91% ±3.98, longer-lived sperm = 23.61% ±4.49; Table 1; Figure 1). This reduction in fertilization success confirms that the manipulation resulted in fewer sperm remaining capable of fertilization, leaving a subset of longer-lived sperm. No difference in larval size was detected between offspring of fresh and longer-lived sperm treatments (Table 1; Figure 1), and thus differences in offspring performance cannot be attributed to variation in offspring size in this study. Intriguingly, larvae from the diluted fresh sperm treatment (which had a similar fertilization success to the longer-lived sperm treatment), were significantly larger than in the fresh sperm treatment (Table 1; Figure 1). In other words, reducing the effective sperm concentration by storing sperm for 1 hour before fertilization had no effect on offspring size, while reducing the sperm concentration by dilution resulted in an increase in offspring size. This implies that the relationship between sperm environment and offspring size in external fertilizers is more complex than previously anticipated. More importantly for interpretation of the results of this study, these results indicate that differences in offspring from fresh versus longer-lived sperm treatments were unlikely to have been driven by differential fertilization of eggs.

**Table 1:** ANOVAs showing the effect of sperm treatment on pre-metamorphic performance.

Source	df	MS	F	P
*a) Fertilization success*				
Treatment	1	2044	6.139	0.023*
Male	19	386	1.159	0.376
Error	19	333		
*b) Hatching success*				
Treatment	1	2250	11.793	0.003*
Male	19	282	1.480	0.200
Error	19	191		
*c) Larval size*				
Treatment	2	2.94	2.891	0.058
Male	4	106	103.885	0.000
Error	185	1.02		
**Post hoc analyses**				
Fresh vs longer-lived sperm	1	0.169	0.166	0.684
Fresh vs diluted sperm	1	5.16	5.068	0.026*

Treatments include fresh sperm versus longer-lived sperm for *(a) fertilization success* and *(b) hatching success*; an additional treatment of diluted fresh sperm is included for *(c) larval size*. *p<0.05.

**Figure 2:**
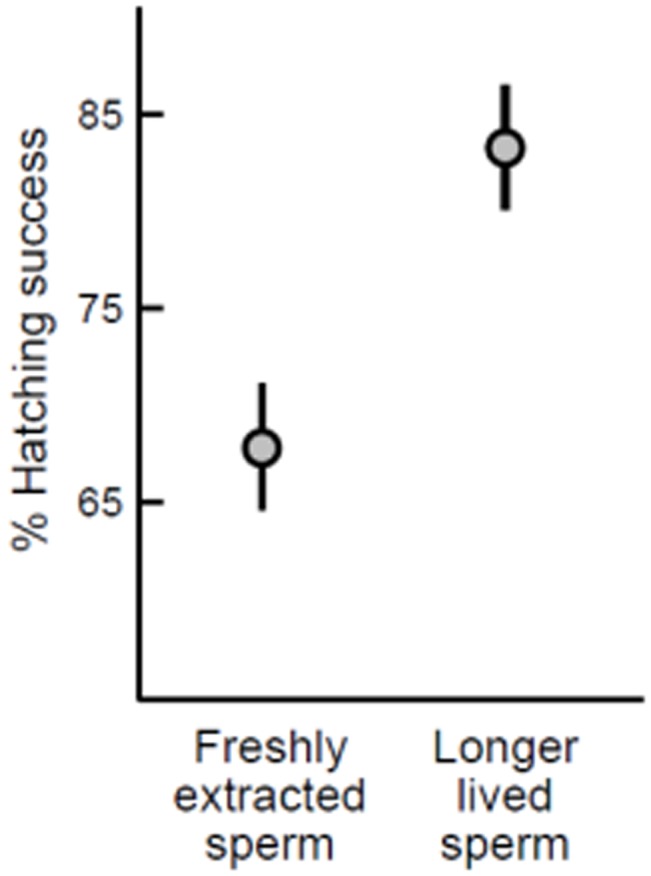
Relationship between sperm longevity and offspring hatching success. Points represent least squares mean (± SE).

**Figure 3:**
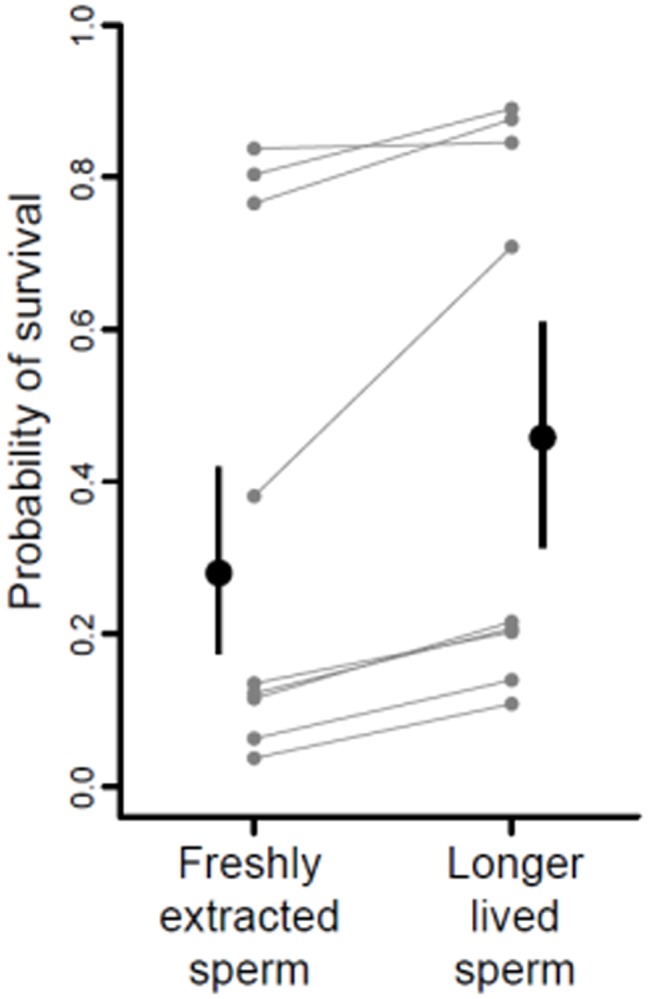
Relationship between sperm longevity and offspring survival for two weeks in the field. Black points represent the back-transformed estimates (± SE) from the mixed-effects logistic regression. Grey points are the estimated mean probabilities for each male (random effect values for each male back-transformed to the probability scale). Grey lines link means from each male.

Eggs fertilized by the subset of longer-lived sperm were more likely to hatch successfully into larvae compared to their siblings that were sired by fresh sperm (Table 1; Figure 2). The link between sperm phenotype and offspring phenotype continued across the metamorphic boundary: offspring sired by longer-lived sperm survived better after two weeks in the field, relative to their siblings sired by fresh sperm (LRT statistic = 9.941, *P* [χ*^2^_3_*] = 0.019; Figure 3). The relationship between sperm longevity and offspring survival was consistent across both low and high density conditions (LRT statistic = 0.490, *P* [χ*^2^_1_*] = 0.484), and therefore the survival advantage of offspring from longer-lived sperm was unaffected by the degree of competition for resources.

## Discussion

A greater proportion of offspring sired by longer-lived sperm hatched into larvae and survived for two weeks in the field, compared to siblings sired by fresh sperm of the same ejaculate. These results indicate that within-ejaculate differences in sperm longevity can affect both pre- and post-metamorphic survival of offspring. We can largely rule out the possibility that this result was driven by maternal effects or cryptic female choice, because *Styela plicata* reproduces by broadcast-spawning. As eggs were harvested from individuals not exposed to experimental treatments and fertilized *in vitro*, females were unable to manipulate which sperm will fertilize their eggs. It is possible that the eggs of some females were better at attracting sperm and therefore these females may have obtained proportionally more fertilizations. However, we believe it is highly unlikely that differential fertilization among females could explain the consistent effects of higher offspring performance in the longer-lived sperm treatment group. For such an effect to drive the results, by chance alone, the different genotypes would have to have consistent ‘preferences’ for fresh or stored sperm and these preferences would have to map to differences in offspring performance. It is also possible that differences in sperm concentration between treatments may have influenced the results, although again this seems unlikely given that we found no difference in offspring size between fresh and longer-lived sperm treatments. Hence, differences in offspring survival appear to be driven by links between sperm and offspring phenotype, with offspring sired by longer-lived sperm showing increased performance compared to offspring sired by freshly-extracted sperm of the same ejaculate.

Links between sperm and offspring phenotypes provides an additional source of variability in offspring which has not previously been considered. Phenotypic traits of one stage may persist due to selection on linked traits expressed in the other life-history stage, possibly creating trade-offs between sperm and offspring performance [27], [28], [29]. For example, increased sperm velocity may have a selective advantage at fertilization, but may come at the cost of reduced offspring fitness, thereby creating trade-offs between these life-history stages. Hence, responses to selection in either or both life-history stages may be constrained, and phenotypes of either life-history stage that appear to be maladaptive may be explained by selection on a linked trait in the other stage [27].

Additionally, selection may act on the amount of within-ejaculate variability in sperm phenotype as a trait in itself. Within-ejaculate variation in sperm phenotype is generally attributed to developmental errors during spermatogenesis and/or poor quality control by the male [30]. Thus, postcopulatory sexual selection is expected to minimise variation in sperm phenotype, with increased risk of sperm competition selecting for the production of an optimal sperm phenotype [8], [12]. However, if sperm phenotype is linked to offspring phenotype, increased within-ejaculate variability in sperm phenotype may be selected for as a bet-hedging strategy. As egg phenotype is linked to offspring phenotype, increased within-brood variation in egg phenotype can have a selective advantage in unpredictable environments by increasing maternal geometric fitness [13], [14]. Further studies are required to determine if within-ejaculate variation in sperm phenotype is under similar selection pressures.

We are unable to determine the mechanism underpinning our results from our current data, but there are several plausible mechanisms that would explain a link between sperm longevity and offspring performance. Given a limited energy budget, there is an expected trade-off between sperm longevity and sperm velocity [31], although this trade-off has not been demonstrated at the intra-male level. Selection pressures on broadcast gametes are very different to internal fertilizers due to the rapid dilution of sperm in the water column [32], [33], [34]. In external fertilizers, sperm half lives range from a few seconds to a few hours, and sperm velocity (in addition to relative sperm abundance) is predicted to be an important determinant of competitive success [31], [35], [36]. Hence, it is possible that high velocity sperm were selected for in the fresh sperm treatment, whereas only slower sperm remained capable of fertilization in the longer-lived sperm treatment. If the metabolic rate of sperm is linked to metabolic rate in offspring, it is possible that differences in metabolic rate could explain differences in offspring performance, however this idea requires further study. Alternatively, if the haploid genome influences sperm phenotype [37], [38], then different sets of alleles may have been selected for in fresh versus longer-lived sperm treatments, and these distinct paternal genomes could have a differential effect on offspring performance. Another intriguing possibility is that selection may have acted on epigenetic differences in gene expression in the sperm [39], [40]. Recent evidence shows that epigenetic marks can be stably transmitted to offspring (e.g. [41], [42]), and therefore it is possible that epigenetic mechanisms could explain transgenerational links between these life history stages [43].

Regardless of the underlying mechanism, this study indicates that within-ejaculate differences in sperm phenotype *can* have transgenerational consequences for offspring performance – indicating that sperm phenotype is linked to offspring phenotype. It has become increasingly apparent that variation in adult fitness may be influenced by embryonic or larval experiences, and that metamorphosis is not a new beginning [44]. Our study extends this link back to sperm phenotype, suggesting that fertilization is not a new beginning.
